# An Emotion-Enriched Context Influences the Effect of Action Observation on Cortical Excitability

**DOI:** 10.3389/fnhum.2017.00504

**Published:** 2017-10-18

**Authors:** Giovanna Lagravinese, Ambra Bisio, Alessia Raffo De Ferrari, Elisa Pelosin, Piero Ruggeri, Marco Bove, Laura Avanzino

**Affiliations:** ^1^Department of Experimental Medicine, Section of Human Physiology, University of Genoa, Genoa, Italy; ^2^Department of Neuroscience, Rehabilitation, Ophthalmology, Genetics and Maternal Child Health, University of Genoa, Genoa, Italy

**Keywords:** emotion, action observation, context, mirror neuron system, transcranial magnetic stimulation

## Abstract

Observing other people in action activates the “mirror neuron system” that serves for action comprehension and prediction. Recent evidence suggests that this function requires a high level codification triggered not only by components of motor behavior, but also by the environment where the action is embedded. An overlooked component of action perceiving is the one related to the emotional information provided by the context where the observed action takes place. Indeed, whether valence and arousal associated to an emotion might exert an influence on motor system activation during action observation has not been assessed so far. Here, cortico-spinal excitability of the left motor cortex was recorded in three groups of subjects. In the first condition, motor-evoked potential (MEPs) were recorded from a muscle involved in the grasping movement (i.e., abductor pollicis brevis, APB) while participants were watching the same reach-to-grasp movement embedded in contexts with negative emotional valence, but different levels of arousal: sadness (low arousal), and disgust (high arousal) (“Context *plus* Movement-APB” condition). In the second condition, MEPs were recorded from APB muscle while participants were observing static images representing the contexts in which the movement observed by participants in “Context plus Movement-APB” condition took place (“Context Only-APB” condition). Finally, in the third condition, MEPS were recorded from a muscle not involved in the grasping action, i.e., abductor digiti minimi, ADM, while participants were watching the same videos shown during the “Context *plus* Movement-APB” condition (“Context *plus* Movement-ADM” condition). Results showed a greater increase of cortical excitability only during the observation of the hand moving in the context eliciting disgust, and these changes were specific for the muscle involved in the observed action. Our findings show that the emotional context in which a movement occurs modulates motor resonance and that the combination of negative valence/high arousal drives the greater response in the observer’s mirror neuron system in a strictly muscle specific fashion.

## Introduction

In daily life we are constantly exposed to people acting in our social world. We are able to describe these actions, to understand their content and to predict their consequences; therefore a link between the agent and the observer must be established. Several studies suggested that humans have a “mirror matching system” (namely the mirror neuron system, MNS) similar to that originally discovered in monkeys (Fadiga et al., 1995; Cochin et al., 1998; Gazzola and Keysers, 2009; Kilner et al., 2009; Mukamel et al., 2010). Whenever we are looking at someone performing an action, beside the activation of visual areas, there is a concurrent activation of the sensorimotor network that is usually recruited when we perform that action (Gallese, 2001). Even if it has been advocated that MNS activity codes only for motor representations and not for actions and does not contribute to action understanding (Hickok, 2009), several neuropsychological (Saygin et al., 2004; Buxbaum et al., 2005; Negri et al., 2007; Tessari et al., 2007; Pazzaglia et al., 2008) and neurophysiological studies (Urgesi et al., 2007a,b) support the general idea that MNS plays a central role in our ability to understand other people’s actions.

In humans, single-pulse transcranial magnetic stimulation (TMS) and related motor-evoked potentials (MEPs) recording during action observation have been largely used to study MNS activity during action observation. Indeed, the amplitudes of MEPs in the contralateral target muscle (that is an index of cortico-spinal excitability) are modulated by action observation, a phenomenon described as “motor resonance” (Fadiga et al., 1995). This ‘motor resonance’ effect is thought to result from activity in MNS regions, which enhances the excitability of the primary motor cortex (M1) via cortico-cortical pathways (Fadiga et al., 2005; Avenanti et al., 2007; Koch et al., 2010; Naish et al., 2014). Indeed, it has been extensively shown that MEPs recorded during action observation are modulated by premotor and parietal regions where mirror neurons have been commonly reported (Avenanti et al., 2007, 2013; Koch et al., 2010) (for an extensive review of TMS-MEPs studies addressing the features of motor resonance see Naish et al., 2014).

Motor resonance is a fundamental component of motor cognition that encompasses how we understand our own movement, and how movement helps us to understand the world. Nevertheless, actions are not perceived in isolation but are context-embedded with objects and actors that create an environment. Recent evidence supported the idea that the comprehension of actions requires a high level codification triggered not only by components of motor behavior, but also by the environment where the action is embedded (Iacoboni et al., 2005; Sartori et al., 2011; Liuzza et al., 2015; Amoruso and Urgesi, 2016). As an example, observing a grasping movement embedded in a context related to movement intention leads to a stronger activation of the MNS when compared to observing the same movement detached from the context or the context alone (Iacoboni et al., 2005). Recently it has been also paid attention to the emotional context where the action is seen (Mazzola et al., 2013; Nogueira-Campos et al., 2016). Observing acts of grasping with the same kinematics but in different emotional contexts (actor performing a grasping with neutral, joyful, or angry expression) could modulate specific brain circuits (Mazzola et al., 2013). Particularly, greater activity in cortical-subcortical network associated with movement control and execution was observed in the anger condition (Mazzola et al., 2013). Further, M1 excitability of the observer’s motor system was influenced by the observation of videos showing a grasping movement directed to emotion-laden objects (Nogueira-Campos et al., 2016). In particular, M1 excitability was higher during the observation of grasping directed to unpleasant compared to pleasant objects (Nogueira-Campos et al., 2016). These findings are in accordance with what reported in the literature on the role of the emotion on the motor system, with negative emotions modulating to a greater extent M1 excitability (Giovannelli et al., 2013; Komeilipoor et al., 2013) and motor behavior (Naugle et al., 2011; Kang and Gross, 2015).

So far, experimental paradigms to test the role of emotional context on action observation were all developed with the aim of testing different valence of emotion (negative vs. positive) with similar level of arousal. This procedure had the aim of disentangling the role of emotional valence from the one of general alertness of the motor system. However, thinking to real life, movement and behavior we daily observe and experience are embedded in a context that can have an emotional valence, influenced by personal past experiences occurred in everyone’s life, but also different arousal. Arousal is a constitutive part of an emotional response in that it influences behaviors, including motor flexibility and action readiness (for a review see Mauss and Robinson, 2009). As an example, startle response is reliably larger in the context of high-arousal negative stimuli and reliably smaller in the context of high-arousal positive stimuli, being not influenced by low-arousal positive or negative stimuli (Lang et al., 1990; Lang, 1995).

Starting from this, we can hypothesize that an emotion-enriched context with negative valence, but different levels of arousal could differently impact motor resonance. We focused here on sadness, which is defined as an unpleasant and low arousal emotion, and disgust, considered an unpleasant and high arousal emotion (Posner et al., 2005). To assess the influence of the emotion-enriched context in the degree of activation of the MNS, M1 excitability was measured by using TMS, during the observation of a similar reach-to-grasp hand movement embedded in a context with negative emotional valence, but different levels of arousal in the observers.

We hypothesized that higher arousal context (disgust) would elicit a higher response of the cortical motor system. Further, we also hypothesized that if level of arousal is another component of the context, in addition to valence, influencing motor resonance mechanisms, then a selective higher excitability during the observation of grasping movement inserted in a disgust context will be recorded in the muscle specifically involved in the grasping movement (APB, abductor pollicis brevis), and not in a muscle not involved in the observed action (ADM, abductor digiti minimi). *Viceversa*, cortical excitability increase will be similar in both APB and ADM muscles if arousal will only provoke a general alertness response.

## Materials and Methods

### Participants

Forty-five healthy subjects (21 males, mean age ±*SD* = 23.18 ± 1.8 years) were enrolled in the study. Subjects with previous or current psychiatric, neurologic or medical diseases were excluded. All participants were naïve to the purpose of the experiment and they gave written informed consent before participation. The experimental protocol was approved by the ethics committee of the University of Genoa and was carried out in agreement with legal requirements and international norms (Declaration of Helsinki, 1964). Right hand dominance was evaluated by the Edinburgh Handedness Inventory (Oldfield, 1971).

### Experimental Paradigm

**FIGURE 1:**
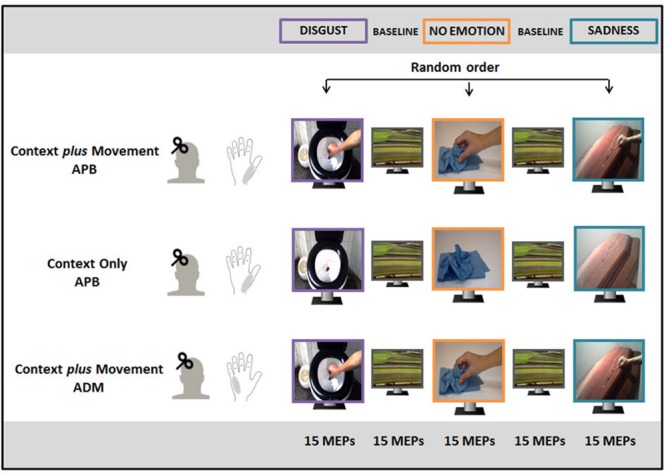
Experimental paradigm. In the “Context *plus* Movement-APB” condition, participants watched a reach-to-grasp movement embedded in two emotional contexts (Disgust and Sadness) and in a No Emotion context and motor-evoked potential (MEPs) were recorded from abductor pollicis brevis (APB) muscle. In the “Context only-APB” condition, participants observed static images representing the three contexts in which the movement observed by participants in the previous condition took place and MEPs were always recorded from APB. In the “Context *plus* Movement-ADM” condition participants observed the same videos used for the “Context *plus* movement-APB” condition but MEPs were recorded from adductor digiti minimi (ADM) muscle.

The experimental paradigm is depicted in **Figure 1**. Subjects were divided into three groups participating to three different experimental conditions: “Context *plus* Movement-APB” condition (fifteen subjects, 7 males, mean age ±*SD* = 23.3 ± 1.8 years), “Context Only-APB” condition (fifteen subjects, 6 males, mean age ±*SD* = 23.2 ± 2 years) and “Context *plus* Movement-ADM” condition (fifteen subjects, 8 males, mean age ±*SD* = 23 ± 1.7 years). In all conditions, subjects were seated on a comfortable chair and were asked to carefully watch videos or images presented on a 19-inch screen located 60 cm from them. Cortical excitability of the left M1 was tested by means of TMS while participants were observing different videos/images appearing on the screen. Each video/image lasted 5 s and the next video was displayed after 3 s during which participants observed a black screen. Each video/image was presented in a block of 15 repetitions, followed by a block of 15 neutral videos (landscape) as baseline.

In the “Context *plus* Movement-APB” condition, three videos of a right hand moving toward an object were filmed and used. Two videos showed a hand reaching different objects in two different emotion-inducing contexts. In particular, they represented a hand grasping a rosary placed on a coffin to elicit sadness (sadness video) and a hand grasping a very dirty toilet paper into a toilet to elicit disgust (disgust video). A third video showed the same movement in a neutral context, i.e., a hand grasping a napkin placed on a table (no-emotion video). Particularly, all movements ended with a precision-grip movement that selectively involves APB muscle activity (Cattaneo et al., 2005; Prabhu et al., 2007; Davare et al., 2009). The order of presentation of the three video blocks was random. The excitability of the cortical area representing the muscle involved in the movement (APB) was studied.

Participants enrolled for the “Context only-APB” condition observed static images representing the three contexts in which the movement observed by participants in the “Context *plus* Movement-APB” condition took place. The order of presentation of static images representing different contexts was randomized. This experiment allowed the evaluation of the effect of the observation of an emotional context *per se*. Again, the excitability of the cortical area representing the muscle involved in the movement (APB) was studied.

Finally, a third group of participants observed the same videos used for the “Context *plus* Movement-APB” condition, but M1 excitability was tested in correspondence to a cortical area representing a muscle not involved in the reach-to-grasp movement, i.e., ADM (Franca et al., 2012) (“Context *plus* Movement-ADM” condition).

At the end of each experimental condition, participants were asked to state the intensity of emotional valence and emotional arousal elicited by the videos using the Self-Assessment Manikin scale (SAM). The SAM is a non-verbal pictorial assessment technique that directly measures the pleasure and arousal associated with a person’s affective reaction to a wide variety of stimuli (Bradley and Lang, 1994). This scale allowed us to collect objective data on subjects’ reactions on emotional videos. In details, it gave us the possibility to measure if subjects perceived the emotional videos as pleasant or unpleasant (considering pleasant something causing a feeling of happiness or pleasure and unpleasant something disagreeable) and to quantify their reaction in terms of arousal.

### Transcranial Magnetic Stimulation (TMS)

Focal TMS was applied on left M1 with a single Magstim 200^2^ magnetic stimulator (Magstim Company) connected with a figure-of-eight coil (wing diameter: 70 mm). The coil was placed tangentially to the scalp with the handle pointing backward and laterally at 45° to the sagittal plane inducing a postero-anterior current in the brain. We determined the optimal position for activation of the right APB muscle in the “Context *plus* Movement-APB” and in the “Context Only-APB” conditions, or of the right ADM in the “Context *plus* Movement-ADM” condition by moving the coil in 0.5 cm steps around the presumed motor hand area. For each participant, the stimulus intensity needed to evoke a MEP of approximately 0.8–1 mV peak-to-peak amplitude was defined (S1mV) was identified at the beginning of the experimental condition. This intensity was used throughout the experimental condition. A custom-made MatLab software managed the synchronization between the presentation of the visual stimulus and the delivering of the magnetic stimulation. In the “Context *plus* Movement-APB” condition and in the “Context *plus* Movement-ADM” condition, the magnetic stimulus was delivered randomly 150 ms before or 150 ms after the contact between the hand and the object. In the “Context Only-APB” condition the magnetic stimulus was delivered randomly while the context was present on the screen. In every experimental session 15 MEPs from the target muscle were collected.

### Electromyographic (EMG) Recording

Electromyographic (EMG) activity was recorded using silver disc surface electrodes. These electrodes were placed over the regions of the APB or ADM muscle belly and associated tendon of the right hand. The ground electrode was placed at the elbow. EMG were digitalized, amplified and filtered (20–1 kHz) with a 1902 isolated pre-amplifier controlled by the Power 1401 acquisition interface (Cambridge Electronic Design Limited, Cambridge, England), and stored on a personal computer for display and later offline data analysis. Each recording epoch lasted 400 ms, of which 100 ms preceded the TMS. Participants were constantly reminded to always keep their hand relaxed during the whole experiment. We cautiously controlled the EMG activity in real-time to ensure that action observation trials were not contaminated by muscle activity. Muscle activity in all trials was lower than 10 μV and not different from muscle activity at rest (*p* > 0.1).

### Data Analysis

Regarding the Self-Assessment Manikin scale administered to participants, following the International Affective Picture System (IAPS) instruction manual (Hillman et al., 2004), we scored the valence and arousal ratings such that 9 represents a high rating on each dimension (i.e., pleasant, high arousal), and 1 represents a low rating on each dimension (i.e., unpleasant, low arousal), to obtain mean ratings for the picture categories. Regarding neurophysiological data, measurements of MEPs were made on single trials. The amplitude of contralateral MEPs (right ABP or right ADM muscles) was evaluated by taking the peak-to-peak difference in the raw EMG signals. Mean values of MEPs amplitude were calculated for each subject, for every video observed, in each experimental condition. Mean values of all MEPs collected during the washout condition were used to create a “Baseline” condition. These MEPs data entered in the analysis as “raw” MEPs data. MEPs data collected during observation of each video were also normalized respect to the “Baseline” condition. These data entered in the analysis as “normalized” MEPs data. Normalization was adopted in order to reduce inter-subjects variability and to test directly for the influence of videos (no-emotion vs. emotional videos) on M1 excitability. No trials were removed from the analysis.

### Statistical Analysis

Regarding the Self-Assessment Manikin scale, arousal and valence ratings on Disgust and Sadness videos collected in all the experimental conditions entered in a RM-ANOVA with Type of Video (Disgust and Sadness) as within-subjects factor and Condition (“Context *plus* Movement-APB, Context Only-APB” and “Context *plus* Movement-ADM”) as between-subjects factor.

Then, to evaluate differences in M1 excitability while subjects where observing the same reach-to-grasp movement performed in different emotional contexts or while they were watching the mere context, raw MEPs data collected from APB muscle in the “Context *plus* movement-APB” condition and in the “Context Only-APB” condition were subjected to a RM-ANOVA with Type of Video (Disgust, Sadness, No-emotion, and Baseline) as within-subjects factor and Condition (“Context *plus* Movement-APB” and “Context Only-APB”) as between subjects factor. A further RM-ANOVA with Type of Video (Disgust, Sadness, No-emotion) as within-subjects factor and Condition (“Context *plus* Movement-APB” and “Context Only-APB”) as between subjects factor, was done on normalized MEPs data to directly test for differences between MEPs amplitude collected during the emotional videos vs. MEPs amplitude collected during the no-emotion video.

Further, to test whether M1 excitability increased selectively during action observation in the muscle involved in the observed action, raw MEPs data obtained from APB muscle in the “Context *plus* Movement-APB” condition were compared to raw MEPs data obtained from ADM in the “Context *plus* Movement-ADM” condition by means of a RM-ANOVA with Type of Video (Disgust, Sadness, No-emotion, and Baseline) as within-subjects factor and Muscle (APB and ADM) as between-subjects factor. Again, a further RM-ANOVA with Type of Video (Disgust, Sadness, and No-emotion) as within-subjects factor and Muscle (APB and ADM) as between-subjects factor, was done on normalized MEPs data to directly test for differences between MEPs amplitude collected during the emotional videos vs. MEPs amplitude collected during the no-emotion video. *Post hoc* analysis was performed by means of Newman–Keuls test. The Bonferroni correction for multiple comparisons was applied and the significance level was set at 0.025. Finally, the Spearman’s correlation coefficient was applied to assess any correlation between MEPs values and valence and arousal scores reported for the emotional videos. Correlation analysis was performed between arousal and valence ratings on Disgust and Sadness videos collected through the Self-Assessment Manikin and changes in M1 excitability during video observation. Normalized MEPs data were entered in the correlation analysis to exclude an effect of inter-subjects baseline variability in cortico-spinal excitability. All statistical analyses were performed using SPSS2.2.

## Results

### Self-Assessment Manikin Ratings

Data from Self-Assessment Manikin from all the participants in the study revealed that, regarding valence, subjects gave a low rating both for Sadness and Disgust videos (meaning unpleasantness). In fact, the statistical analysis showed no significant differences for the Type of video [*F*(1,26) = 0.72, *p* = 0.41] nor for the interaction Type of Video ^∗^ Condition [*F*(2,26) = 0.09, *p* = 0.91]. Concerning arousal, the RM-ANOVA showed a significant effect of Type of Video [*F*(1,26) = 64.7, *p* = 0.0001] and no significant interaction Type of Video ^∗^ Condition [*F*(2,26) = 0.48, *p* = 0.63]. All subjects gave a lower rating for Sadness video with respect to Disgust video (*p* = 0.0001) (**Table 1**).

**Table 1:** Mean intensity of emotional valence and emotional arousal (Self-Assessment Manikin ratings) perceived by subjects in the “Context *plus* Movement-APB” condition, “Context Only-APB” condition and in the “Context *plus* Movement-ADM” condition.

	Context *plus* Movement-APB		Context Only-APB		Context *plus* Movement-ADM		
	Sadness	Disgust	Sadness	Disgust	Sadness	Disgust	*p*-level
Valence	2.05 ± 0.68	1.9 ± 0.88	2.23 ± 1.01	1.84 ± 0.91	2.16 ± 0.75	2 ± 0.63	*p* > 0.05
Arousal	4.25 ± 1.93	7.2 ± 0.75	4 ± 1.41	6.43 ± 1.51	2.89 ± 1.16	6.83 ± 1.83	*p* = 0.0001

*Values are presented as mean ± SD.*

### MEPs Results

The neurophysiological data showed that, in the “Context *plus* Movement-APB” condition the emotional context in which action was embedded influenced the motor resonance. In particular, the video eliciting disgust was the most effective in increasing M1 excitability during action observation. Statistical analysis (RM-ANOVA) showed a significant interaction Type of Video ^∗^ Group [*F*(3,84) = 9.13, *p* = 0.00002]. As expected, *post hoc* analysis showed that in the “Context *plus* movement-APB” condition, MEPs collected in the Baseline were significantly lower than MEPs collected while subjects were observing all the videos showing the grasping movement (Baseline vs. No-emotion, *p* = 0.009; Baseline vs. Sadness, *p* = 0.0002; Baseline vs. Disgust, *p* = 0.0001).

**FIGURE 2:**
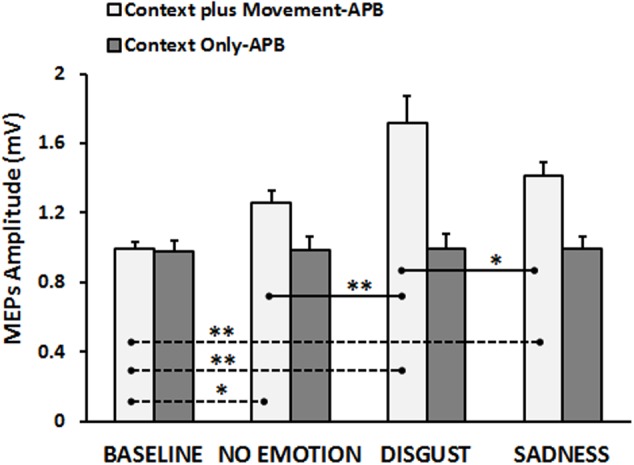
Cortico-spinal excitability of the left hemisphere evaluated during the observation of different types of videos in the “Context *plus* Movement-APB” and in the “Context Only-APB” conditions. *Y*-axis represents the MEPs amplitude (mV). Dashed black lines indicate when baseline video was significantly different from other conditions, whereas straight black lines indicate that Disgust video was significantly different from the No-emotion and Sadness grasping conditions. Vertical bars indicate SE. Asterisks indicate the level of significance (^∗^*p* < 0.025, ^∗∗^*p* < 0.001).

*Post hoc* test showed also that only MEPs collected while participants were observing the Disgust video were significantly higher than MEPs collected when subjects were observing the No-emotion video (Disgust vs. No-emotion: *p* = 0.0001) and the Sadness video (Disgust vs. Sadness: *p* = 0.002), whereas there was no difference between MEPs collected during the observation of the Sadness video and the grasping No-emotion video (*p* = 0.11). Differently, in the “Context Only-APB” condition, *post hoc* analysis showed that when participants observed the mere context no significant differences among videos appeared (No-emotion vs. Sadness, *p* = 0.99; No-emotion vs. Disgust, *p* = 0.95; Disgust vs. Sadness, *p* = 0.96). Results are shown in **Figure 2**. These results were confirmed by the statistical analysis on normalized data. Indeed, the RM-ANOVA showed a significant effect of Type of Video [*F*(2,56) = 4.40, *p* = 0.017] and a significant Type of Video ^∗^ Group [*F*(2,56) = 4.62, *p* = 0.014] interaction. *Post hoc* analysis revealed that only in the “Context *plus* movement-APB” condition, MEPs collected when subjects were observing the Disgust video were significantly higher than MEPs collected when subjects were observing the No-emotion (*p* = 0.001) and Sadness (*p* = 0.015) videos, with no difference between No-emotion and Sadness (*p* = 0.08). In the “Context *only*-APB” condition, there was no difference between the MEPs collected when subjects were observing the No-emotion and both the Disgust (*p* = 0.85) and the Sadness (*p* = 0.77) videos and no difference between MEPs recorded during the Disgust and the Sadness (*p* = 0.99) videos.

**FIGURE 3:**
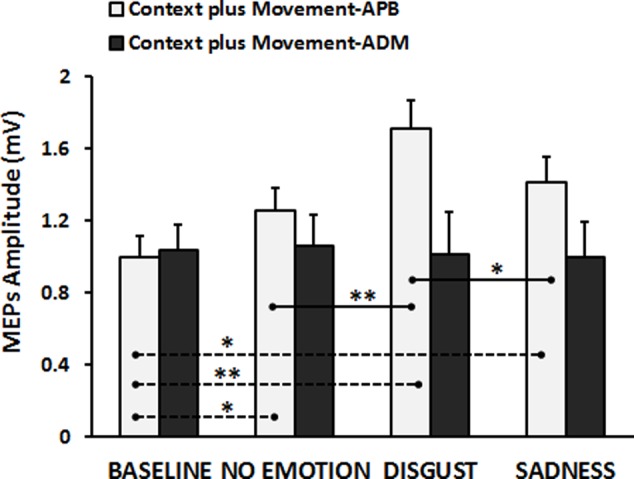
Cortico-spinal excitability of the left hemisphere evaluated during the observation of different types of videos in the “Context *plus* Movement-APB” and in the “Context *plus* Movement-ADM” conditions. *Y*-axis represents the MEPs amplitude (in mV). Dashed black lines indicate when baseline video was significantly different from other conditions, whereas straight black lines indicate that Disgust video was significantly different from the No-emotion and Sadness grasping conditions. Vertical bars indicate SE. Asterisks indicate the level of significance (^∗^*p* < 0.025, ^∗∗^*p* < 0.001).

Finally, the results of the RM-ANOVA comparing “Context *plus* Movement-APB” and “Context *plus* Movement-ADM” conditions revealed a significant interaction Type of Video ^∗^ Muscle [*F*(3,84) = 8.46, *p* = 0.00005]. *Post hoc* test showed that the context in which the grasping movement was inserted was effective in modulating the excitability only of the cortical representation of the muscle involved in the observed movement, i.e., APB muscle, confirming the results presented in the previous analysis (Baseline vs. No-emotion, *p* = 0.024; Baseline vs. Sadness, *p* = 0.004; Baseline vs. Disgust, *p* = 0.0001; Disgust vs. No-emotion: *p* = 0.0001; Disgust vs. Sadness: *p* = 0.006), whilst no significant effect was found when MEPs were recorded from ADM muscle (*p* always > 0.05) (**Figure 3**). These findings were confirmed by the statistical analysis on normalized data. Indeed, the RM-ANOVA showed a significant effect of Muscle [*F*(1,28) = 15.42, *p* = 0.001], indicating that MEPs amplitude changed in a strictly muscle-specific fashion only in APB muscle during observation of a precision-grip movement. Further, a significant Muscle ^∗^ Type of Video interaction emerged [*F*(2,56) = 3.89, *p* = 0.026]. *Post hoc* analysis showed that MEPs recorded from APB muscle during the observation of the Disgust were significantly higher than MEPs recorded during the observation of the No-emotion (*p* = 0.001) and the Sadness (*p* = 0.018) videos, whereas no difference emerged between the No-emotion and the Sadness (*p* = 0.36) videos. Differently, MEPs recorded from ADM muscle during the observation of the three videos did not differ between them (Disgust vs. No-emotion, *p* = 0.68; Sadness vs. No-emotion, *p* = 0.74; Sadness vs. Disgust, *p* = 0.58).

### Correlation Analysis

**FIGURE 4:**
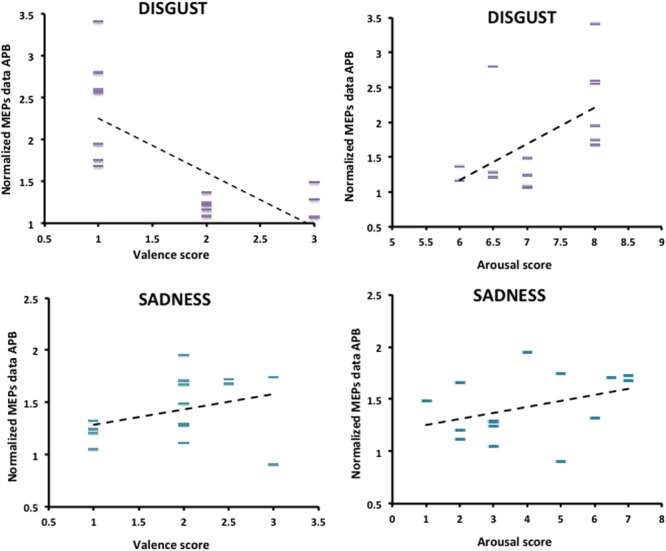
Correlation between arousal and valence ratings on Disgust and Sadness videos collected through the Self-Assessment Manikin and changes in the excitability of the M1 area representing the APB muscle, during video observation. Valence and Arousal scores are showed on the *X*-axis. MEPs data collected during the observation of the emotional videos (Disgust and Sadness) are normalized respect to MEPs data collected at baseline and are showed on the *Y*-axis.

The relationship between normalized MEPs values recorded from APB muscle in the “Context *plus* Movement-APB” condition while observing the different emotional videos and valence and arousal scores collected through the Self-Assessment Manikin showed a significant negative correlation with Disgust valence scores (Spearman’s rho = -0.70, *p* < 0.001) and a significant positive correlation with Disgust arousal scores (Spearman’s rho = 0.58, *p* = 0.02) (**Table 2** and **Figure 4**). These findings indicate that the more the MEPs recorded during the observation of the video eliciting Disgust were higher, the more the valence score was lower and the arousal score was higher, respectively. No significant correlations were found for the Sadness video (**Table 2** and **Figure 4**). Further, no significant correlations were found between Disgust valence and arousal scores (**Table 2**) and between Sadness valence and arousal scores (**Table 2**) when MEPs were collected from ADM muscle.

**Table 2:** Correlations between normalized MEPs (MEPs video/MEP baseline) collected during the observation of the emotional videos from APB muscle in the “Context *plus* movement-APB” condition and from the ADM muscle in the “Context plus movement-ADM” condition and valence ad arousal scores from Self-Assessment Manikin.

	Valence		Arousal	
	Spearman rho	*p*-level	Spearman rho	*p*-level
**Context *plus* Movement-APB**				
Sadness	0.45	0.09	0.44	0.09
Disgust	-0.70	<0.001^∗∗^	0.58	0.02^∗^
**Context *plus* Movement-ADM**				
Sadness	0.09	0.76	0.009	0.97
Disgust	0.09	0.73	0.39	0.14

*Asterisks indicate when correlation analysis yielded a significant difference (^∗^*p* < 0.05, ^∗∗^*p* < 0.01).*

## Discussion

In this study we tested cortico-spinal reactivity to TMS, an index of motor resonance, during observation of videos of the same grasping movement but embedded in different emotional contexts. In particular, we focused the attention on contexts able to evoke two emotions with the same negative valence, but with different levels of arousal.

The results showed that the observation of a hand moving in a context eliciting unpleasant and high arousal reaction (Disgust) was the only one able to increase the cortical excitability more than the observation of the same hand movement in a no-emotion, neutral context. Indeed, when subjects observed the hand moving in a context eliciting unpleasant but low arousal reaction (Sadness), motor resonance was similarly modulated as when subjects observed the hand movement in a no-emotion, neutral context. Further, this effect was specifically related to mirroring the observed action and not due to less specific modulations of the motor system, since these changes were specific for the muscle involved in the observed action. Related only to Disgust, we also found that the more the observer’s cortical excitability increased, the more the observer gave a low rating on the unpleasant/pleasant dimension and a high rating on the low arousal/high arousal dimension. Finally, the observation of the mere emotional-inducing contexts, without the hand movement, was not able to induce changes in motor resonance.

Our study enters in a well-established scenario that links emotions, and particularly negative emotions, to the activity of the motor system. Indeed, research based on audio-motor or visuo-motor coupling showed that negative emotions exerted a stronger effect on the motor system than neutral conditions or positive emotions. Giovannelli et al. (2013) showed that fear-related music significantly increased the M1 excitability compared to the neutral piece. Notably, this effect was not observed with music pieces inducing other emotional experiences. Similarly, it has been showed a significant increase in cortico-spinal excitability in response to unpleasant sounds (such as explosion, siren, man sobbing, buzzing, dentist drill) as compared to neutral sounds (Komeilipoor et al., 2013). More recently, by using images selected from the IAPS (Lang et al., 2008), Borgomaneri et al. (2014) showed that negative stimuli are able to increase cortico-spinal excitability even in an immediate phase of the stimulus presentation (150 ms after stimulus onset), whilst both positive and negative stimuli induce motor facilitation in a later phase (300 ms after stimulus onset). Actually, it is worth to mention that the effect of arousal/negative/threatening scenarios on M1 is not always facilitatory. Some studies showed an initial inhibitory response to threatening stimuli (Lang and Bradley, 2010; Hagenaars et al., 2014). It must be reported that this transient “freezing like” response occurred when MEPS were recorded at very early latencies (∼100–150 ms after stimulus onset) but not at later timing (Borgomaneri et al., 2015a,b, 2017). Our study, however, focuses on the motor resonance effect, a later phenomenon which is expected to be influenced in an excitatory manner by arousing and negative stimuli (Mazzola et al., 2013; Nogueira-Campos et al., 2016).

Starting from the “circumplex model of affect,” which states that a person’s affective state arises from two neurophysiological systems, one related to valence (a pleasure-displeasure continuum) and the other to arousal or alertness (Russell, 1980), in the last years some attention has been devoted to the combined effects of arousal and valence on motor system. Kang and Gross (2015) showed that the low arousal unpleasant emotion of sadness slowed down a sit-to-walk task, whilst the high arousal emotions of anger and joy speeded it up. Similarly, exposure to highly arousing unpleasant pictures reduced reaction times of gait initiation compared to other affective conditions (low arousing pleasant, high arousing pleasant, low arousing unpleasant) (Naugle et al., 2011).

Related to the mirror neuron system, it has been demonstrated that the emotional state of the observer exerts an influence on the MNS activation. Indeed, when a subject was primed with an emotionally negative picture taken from the IAPS (Lang et al., 2008) the MNS was more responsive to a neutral action (Enticott et al., 2011, 2012; Hill et al., 2013) with respect to when priming was done with emotionally positive pictures. However, in these studies, MNS activity has been explored during the observation of movement taking place in a “neutral” context; i.e., a context lacking of emotional valence, and the sole priming effect of emotional states on MNS activity has been proven. More recently, Mazzola et al. (2013) showed that when the context in which the observed action was performed contained emotional information, as when observing acts of grasping performed by an actor with neutral, joyful, or angry expression, greater activity in cortical–subcortical network associated with movement control and execution was observed in the unpleasant condition with respect to the pleasant or neutral. In addition, Nogueira-Campos et al. (2016) assessed the excitability of the observer’s motor system during action observation of a grasping movement directed to emotion-laden objects. Again, objects inducing negative emotion exerted a higher influence over the MNS activity compared to positive.

However, so far, the influence of the arousal degree of negative emotions on MNS was not considered. Indeed, in accordance with Posner’s theory on emotions (Posner et al., 2005), we paid attention not only on the valence dimension of emotion, focusing on negative valence, but also on different levels of arousal. We found that only the high arousal unpleasant Disgust-inducing grasping video increased more the cortico-spinal excitability than a neutral grasping video, whereas the low arousal unpleasant grasping Sadness-inducing video did not. Our findings strongly recall what it is commonly observed for another emotionally driven motor response that is the startle response. Startle in response to a sudden, intense stimulus is a universal reflex that involves multiple motor actions, including tensing of the neck and back muscles and an eye blink (Landis and Hunt, 1939). The startle response serves a protective function, guarding against potential bodily injury (particularly of the eye) and serving as a behavioral interrupt that is thought to facilitate vigilance in relation to a possible threat (Graham, 1979). Picture-evoked affects have been shown to modulate responses to startle probe stimuli. Indeed, startle reflexes were potentiated during exposition to unpleasant pictures and inhibited during pleasant pictures (Lang, 1995; Cuthbert et al., 1996), and both effects were augmented by high picture arousal. This result is in line with the idea that arousal is the first, necessary step in activating defense behavior in both animals and humans (for a review see Kozlowska et al., 2015). Indeed, when the avoidance system is activated by a negative emotional state, then defensive responses (including the startle reflex) should be primed and thus increased relative to during neutral states. Related to our findings, we can speculate that the observed increase in motor resonance might be the consequence of a reaction of our motor system to a situation from which it is better to escape or to avoid. Observing an action in an emotion-enriched context that provokes unpleasant and high arousal reaction (disgust) may accentuate the mirror neuron system activity in order to plan a faster and more accurate reaction. This hypothesis is also supported by the linear relationship we found between valence, arousal, and motor resonance: the more the subjects perceived the disgust video as unpleasant (valence) and felt activated by the video in terms of alertness (subjective arousal) the more the response of the motor system was high.

Interestingly, we observed that the increase in the response of the motor system during observation of an action taking place in a negative valence/high arousal context was specific for the muscle directly involved in the observed action. Indeed, we only found a higher cortical excitability compared to baseline for APB but not for ADM muscle. It has already been shown that, during action observation, as well as during action execution, APB muscle activity is involved in precision grip (as tested here) and in whole hand grasp, whereas ADM muscle is involved in a whole hand grasp, but not in a precision grip (Cattaneo et al., 2005; Prabhu et al., 2007; Davare et al., 2009). The specific increase of the cortical excitability associated to APB muscle area here shown could appear in contrast with the physiological reaction of general activation caused by the defense cascade consisting of autonomic-driven physiological changes, such as acceleration of cardiac and respiratory rates, release of stored energy, dilation and more blood supply to muscles, mydriasis and so forth (Cannon, 1929). However, we may speculate that even if a generalized response to an emotional stimulus may occur during the observation of an action taking place in a high arousal/negative valence context, this response may be primarily modulated by the motor resonance mechanisms induced by action observation. Since it is known that motor resonance is selective for the muscles involved in the observed action (Grafton and Hamilton, 2007), it is on these specific muscles (the APB in our case) that this reaction exerts its effect, suggesting that even when mirror system activity is influenced by the valence and the arousal of an emotional context, the observed movements are processed in a strictly muscle specific fashion.

Our data well fit with the general idea that an important purpose of the MNS is to respond in real time and in a socially appropriate fashion to the actions of others, rather than just simply understand or predict other people’s action (Hamilton, 2013). Accordingly, MNS engagement has been proven to be driven by social reciprocity (Sartori et al., 2012) and social intention (Liuzza et al., 2015). Interestingly, Liuzza et al. (2015) went further by demonstrating that individual differences in personality traits modulated MNS activity. MNS activity was suppressed during immoral actions observation only in those individuals who exhibited high scores in harm avoidance, a personality trait characterized by excessive worrying and fearfulness (i.e., people more vigilant toward social cues which convey information about potential danger or harm). Potential limitations of the present study need to be mentioned. We did not explore here whether there is a correlation between the individual emotional empathy ability (that can be tested through ad hoc questionnaires) and the degree of activation of the MNS during action observation in an emotion-enriched context. It will be interesting in future studies to enroll a larger number of participants, to characterize them in terms of anxiety and depressive traits and emotional empathy abilities in order to find out whether personality traits influence the way the motor resonance is modulated by the emotion-enriched context where an action takes place. Another limitation concerns some specific features of the contexts that could influence the observers, like visual perspective and the objects being grasped. In fact, the visual perspective of the action (Maeda et al., 2002) and object weight (Alaerts et al., 2010; Tidoni et al., 2013; Valchev et al., 2015) can affect motor resonance mechanism. Nevertheless, we preferred to keep these subtle differences in order to favor the ecological appearance of the action. Finally, a potential limitation of the study is the use of between-subject design. However, we adopted a between-subjects design to avoid a carryover effect when testing changes in M1 excitability during observation of hand moving in a context or of the context only.

## Conclusion

We confirmed the hypothesis that the human mirror neuron system does not simply provide an action recognition mechanism. Rather, motor resonance seems to be largely influenced by top-down components that make the neural system able for coding not only the intentions of others (Iacoboni et al., 2005), but also the emotions of others, taking into account both valence and arousal of the emotion. Interestingly, the modulation consisted in a muscle-specific increase in cortico-spinal excitability when participants observed a grasping movement inserted in a highly arousing unpleasant context. We speculated that the combination of negative valence/high arousal in the context where the movement takes place might drive an increase in motor resonance as a defensive behavior, however, strictly modulated by one of the rules of motor resonance that is muscle-specificity. Future studies are needed to confirm this hypothesis.

## Author Contributions

LA, GL, and EP: Conceived and designed the experiments. AF, GL, and AB: Performed the experiments. EP, AF, and AB: Analyzed the data. GL, LA, AB, PR, and MB: Wrote the paper. LA, MB, and EP: Interpreted the data. LA, AB, GL, and EP: Drafted the article. GL, LA, AB, MB, and PR: Critically revised the article for important intellectual content.

## Conflict of Interest Statement

The authors declare that the research was conducted in the absence of any commercial or financial relationships that could be construed as a potential conflict of interest.
